# Interprofessional socialization of first-year medical and midwifery students: effects of an ultra-brief anatomy training

**DOI:** 10.1186/s12909-024-05451-w

**Published:** 2024-04-26

**Authors:** Dana Bostedt, Ebrar Hümeyra Dogan, Sina Chole Benker, Maret Antje Rasmus, Emily Eisner, Nadine Lana Simon, Martina Schmitz, Markus Missler, Dogus Darici

**Affiliations:** 1 Medical faculty, Universität Münster, 48149 Münster, Germany; 2 Institut für Anatomie und Vaskuläre Biologie, Universität Münster, 48149 Münster, Germany; 3 Institut für Anatomie und Molekulare Neurobiologie, Universität Münster, Vesaliusweg 2-4, 48149 Münster, Germany

**Keywords:** Interprofessional education, Interprofessional socialization, Interprofessional identity, Anatomy education, Ultra-brief anatomy training

## Abstract

**Background:**

Interprofessionalism is considered a key component in modern health profession education. Nevertheless, there remains ongoing debate about when and where to introduce interprofessional trainings in the curriculum. We identified anatomy, a subject commonly shared among health professionals, as a practical choice for initiating early intergroup-contact between first-year medical and midwifery students. Our study examined the effects of a four-hour block course in anatomy on interprofessional socialization and valuing, as well as long-term effects on intergroup contact.

**Methods:**

Based on different concepts and theories of learning, we implemented 12 interprofessional learning stations. Several measures were taken to foster group cohesion: (1) self-directed working in interprofessional tandems on authentic obstetric tasks, (2) competing with other tandems, (3) creating positive interdependencies during task completion, and (4) allowing room for networking. In a pre-post design with a three-month follow-up, we assessed the outcomes of this ultra-brief training with qualitative essays and quantitative scales.

**Results:**

After training, both groups improved in interprofessionalism scores with strong effect sizes, *mean* difference in ISVS-21 = 0.303 [95% CI: 0.120, 0.487], *P* < .001, η² = 0.171, while the scales measuring uniprofessional identity were unaffected, *mean* difference in MCPIS = 0.033 [95% CI: -0.236, 0.249], *P* = .789. A follow-up indicated that these positive short-term effects on the ISVS-21 scale diminished after 12 weeks to baseline levels, yet, positive intergroup contact was still reported. The qualitative findings revealed that, at this initial stage of their professional identity development, both medical and midwifery students considered interprofessionalism, teamwork and social competencies to be of importance for their future careers.

**Conclusion:**

This study advocates for an early implementation of interprofessional learning objectives in anatomical curricula. Young health profession students are receptive to interprofessional collaboration at this initial stage of their professional identity and derive strong advantages from a concise training approach. Yet, maintaining these gains over time may require ongoing support and reinforcement, such as through longitudinal curricula. We believe that an interprofessional socialization at an early stage can help break down barriers, and help to avoid conflicts that may arise during traditional monoprofessional curricula.

## Background

*Interprofessional education* (IPE) is a learning approach in which two or more professions “*learn with, from, and about each other to improve collaboration in patient care*” (WHO 2010). Some of the IPE goals are to exchange ideas on a personal level, bring together distributed knowledge, and harness the benefits of different specializations [1–3]. By fostering interprofessional communication and teamwork, IPE has been shown to break hierarchical structures [2], reduce stereotypes in health professions education [3], and ultimately improve patient care and job satisfaction [4]. 

Despite the chances IPE offers, there is still an ongoing debate on *when* and *where* to implement IPE into existing health profession curricula. One administrative and logistical challenge of IPE is that aligning schedules involves coordinating with multiple faculties, finding common time slots, and ensuring that students can participate without disrupting their core learning objectives. Nonetheless, proponents argue that early implementation of IPE, despite the scarcity of available time slots, is favorable. Thus, implementing IPE in undergraduate health profession curricula may promote early interprofessional socialization between various professions and effectively counteract stereotypes between them [2]. 

To address this feature of IPE, we identified anatomy as a feasible subject to enable early intergroup contact between undergraduate, first-year medical and midwifery students [5–10]. Following socio-constructivist principles of learning [1], which sees individual knowledge as socially constructed, we designed and implemented an interprofessional block training. We put effort to embody these principles through (a) the emphasis on collaborative learning and problem-solving activities among students in interprofessional tandems, (b) through authentic and contextual learning stations within real-world healthcare scenarios, and (c) through facilitating reflection and networking events.

In a mixed-methods approach, we explored the short- and long-term effects of this novel training on interprofessional socialization and valuing, as well as interprofessional identity and intergroup contact. We conclude by evaluating the challenges and merits of this new approach and derive recommendations for future curriculum planners.

### Theoretical introduction into related constructs and IPE theories

#### Interprofessional socialization and valuing

According to Arnold et al. (2020) [11], health professionals usually go through monoprofessional socialization during their training period. As a result, professionals work side by side, but not necessarily with each other, in their daily professional lives. Thus, interprofessional socialization is necessary not only to ensure better communication but also to prevent the isolation of professional groups [3, 12]. This enables students to build a dual, professional and interprofessional, identity. Khalili et al. (2013) [13] suggest a three-stage process when transforming from an uniprofessional identity to a dual professional and interprofessional identity, (1) breaking down barriers, (2) interprofessional tole learning and interprofessional collaboration, and (3) dual identity development. We argue that an early exposure to IPE may break down barriers because it helps to shape perceptions and attitudes before monoprofessional identities become deeply ingrained.

#### Interprofessional identity (IPI)

Through effective interprofessional socialization, students are likely to develop an interprofessional identity. IPI can be understood as a sense of belonging in one’s own professional group to an interprofessional community [13, 14]. IPI assists students in expanding their monoprofessional perspective of practice to encompass a wider interprofessional viewpoint that appreciates the contributions of other professions to client care [3, 15, 16]. Education focusing solely on uniprofessional identity formation bears the risk of dis-integrating one’s own profession from interprofessional elements [2]. At the same time, interprofessional and professional identities are intertwined and influence each other [3]. The establishment of a strong IPI is thought to be particularly beneficial because it increases efficiency and appreciation within the team [12]. 

#### Intergroup contact

Interprofessional socialization is reinforced by intergroup contact [13]. The *contact hypothesis* [17] suggests that bringing together different groups can effectively reduce prejudice between the group members. Constant intergroup contact may contribute to an interprofessional identity as different groups get to know each other better, build relationships, and become more positive about working together [2]. Feeling a sense of belonging to a group is a significant requirement in the professional realm and appears essential for cultivating a positive work environment. When extended to an interprofessional setting, fostering a favorable disposition toward interprofessionalism becomes possible [3]. 

The current study made a concerted effort to integrate these theoretical underpinnings into both the overall design of the training and the specific setup of the training stations, as detailed in the methods section.

### Research questions and hypotheses

We asked whether an ultra-brief interprofessional training in anatomy (i.e., a four-hour block) may be sufficient to promote key elements of interprofessionalism. Specifically, we hypothesized that an ultra-brief anatomy training would have H1: positive effects on interprofessional valuing and socialization, and H2: intergroup contact (primary outcomes). We further hypothesized that these effects would be measurable H3: directly after training, and, with the anticipation that the positive effects of this training would persist beyond the classroom setting through ongoing intergroup contact, H4: three months after training (secondary outcomes). Finally, we explored professional and interprofessional identity formation in medical and midwifery students and conclude with practical implications for future curriculum design.

## Materials and methods

A pre-post interventional study with a three-month follow-up was conducted at the University of Münster, Germany, during the winter term of 2022/2023. The study protocol was deemed not to require formal medical ethics approval. Study participation was voluntary, and informed consent was received from all participants. Data generated or analyzed during the study can be requested from the corresponding author.

### Overview of the medical curriculum

The medical program at the University of Münster is a six-year curriculum that combines theoretical university teaching with practical training in hospitals. The final exam is the state medical examination, which is divided into two sections after 4 and 10 semesters of studying. The four-semester preclinical section focuses on fundamental knowledge of the human body and the natural sciences. Students attend lectures, seminars and practical courses in biology, chemistry, physics, biochemistry, physiology and anatomy. Sociology and psychology subjects teach the theoretical foundations of the doctor-patient relationship and communication. The preclinical section concludes with the preliminary exam. The clinical section of the curriculum consists of topic-based subject modules and block practical courses that introduce students to different medical specialties. Complementary courses cover communication skills, ethics, and the doctor-patient relationship. The clinical section concludes with the second medical license examination, the final clinical year, and the third medical license examination.

### Overview of the midwifery curriculum

The Midwifery Bachelor of Science program at the University of Münster is a dual, primarily professionally qualifying program that combines practical professional training with university teaching and academic education. The standard period of study is 8 semesters with a volume of 240 ECTS (European Credit Transfer and Accumulation System) points. The curriculum is structured to provide knowledge and skills (in various aspects) from the physiological basics to norm variants to pathology around pregnancy, birth and the first time as a family. In the first semesters, students acquire evidence-based midwifery knowledge and skills in the areas of the physiology of pregnancy, childbirth, and the postpartum period, as well as in the physiological development of the newborn and infant. After that, students learn about pathological conditions in these areas. Important practical skills and hand movements are practiced in the practical exercises at the “study hospital”. In addition, the teaching of scientific work, communication skills, midwifery and health care research, as well as ethics and professional policy round out the acquisition of competencies in the profession of midwifery.

### Bringing together anatomy for medical and midwifery students

Both, medical and midwifery students study anatomy in their first year. Medical students have mandatory anatomy lessons, four hours weekly in the first term, 7.7 h in the second, and 8.3 h in the third term. The anatomical curriculum proceeds as follows: The first semester covers general anatomy and embryology through theoretical introductions. The second semester includes seminars and practical instructions, focusing on macroscopic anatomy and a full dissection course. In the third semester, students delve into histology and neuroanatomy, concluding with a four-day anatomy and imaging block course. To complete the anatomy course, students must pass written and oral exams.

Midwifery students, in the first semester, integrate anatomy into their curriculum through a module called ‘basic sciences,’ dedicating four hours weekly. The anatomy coursework emphasizes general embryology and the reproductive system. This teaching approach includes histology, sonoanatomy, and in-depth gross anatomy sessions using prosections, i.e. using prepared cadavers to demonstrate anatomical structures. The primary goal is to equip students with proficiency in medical terminology and the ability to connect their anatomical knowledge to physiological processes in the human body. At the semester’s end, students undergo assessment via a written and an oral exam.

### The design of an ultra-brief interprofessional training in anatomy

**Fig. 1:**
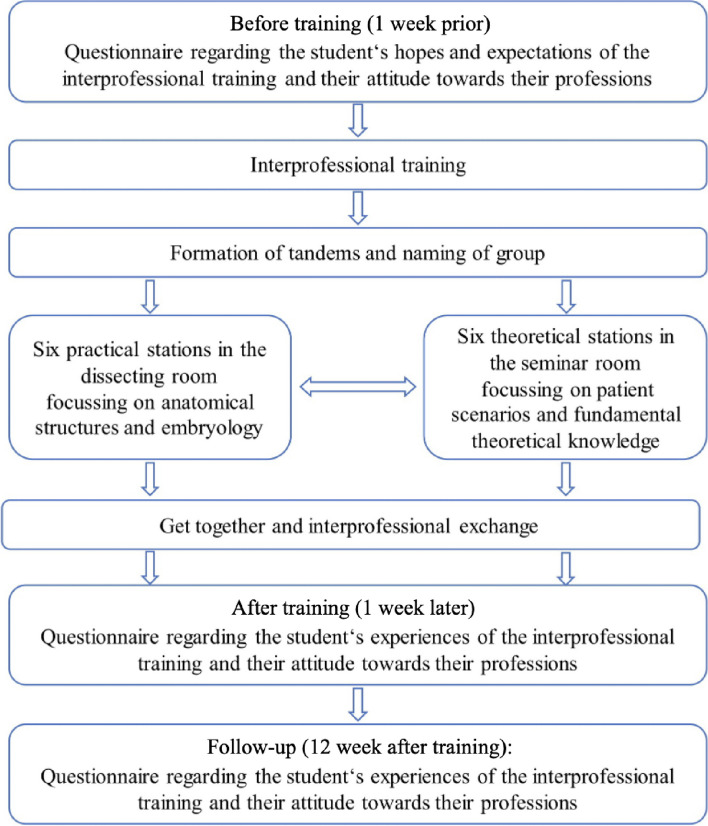
Flowchart of the ultra-brief interprofessional training in anatomy

After a brief welcome and an overview of the upcoming four-hour interprofessional training, midwifery science and medical students formed self-selected groups of two or three, consisting of students from both professions (Fig. 1). The “selection” was based on openness and a positive first impression, with no specific order or pattern. To strengthen the interprofessional identity, the tandems gave themselves their own names and competed with each other against the other tandems.

The 18 interprofessional tandems were divided into two large groups. One group went through six theoretical stations covering hormones and diagnoses, while the other group completed six tasks related to body donations and various models in the dissection room. After their initial stations, they swapped places, resulting in each tandem experiencing a total of twelve stations, each lasting 15 min. In these interprofessional tandems, students mastered anatomical case studies, played “hormone memory”, solved tests on body donations, discussed embryology topics, and assessed spermiograms under microscopes. The use of authentic healthcare scenarios aligns with socio-constructivst theories that advocate for learning in context. Students managed task distribution and documentation themselves. Afterward, there was a joint meal, providing an opportunity to connect, network and reflect on their shared experiences in the medical field to create intergroup contact. Additionally, several students proposed the creation of a shared WhatsApp group to stay in touch. Participation in the group was optional, yet all students chose to engage with this platform for exchange to establish long-term intergroup contact.

### Details on the interprofessional training stations

**Fig. 2:**
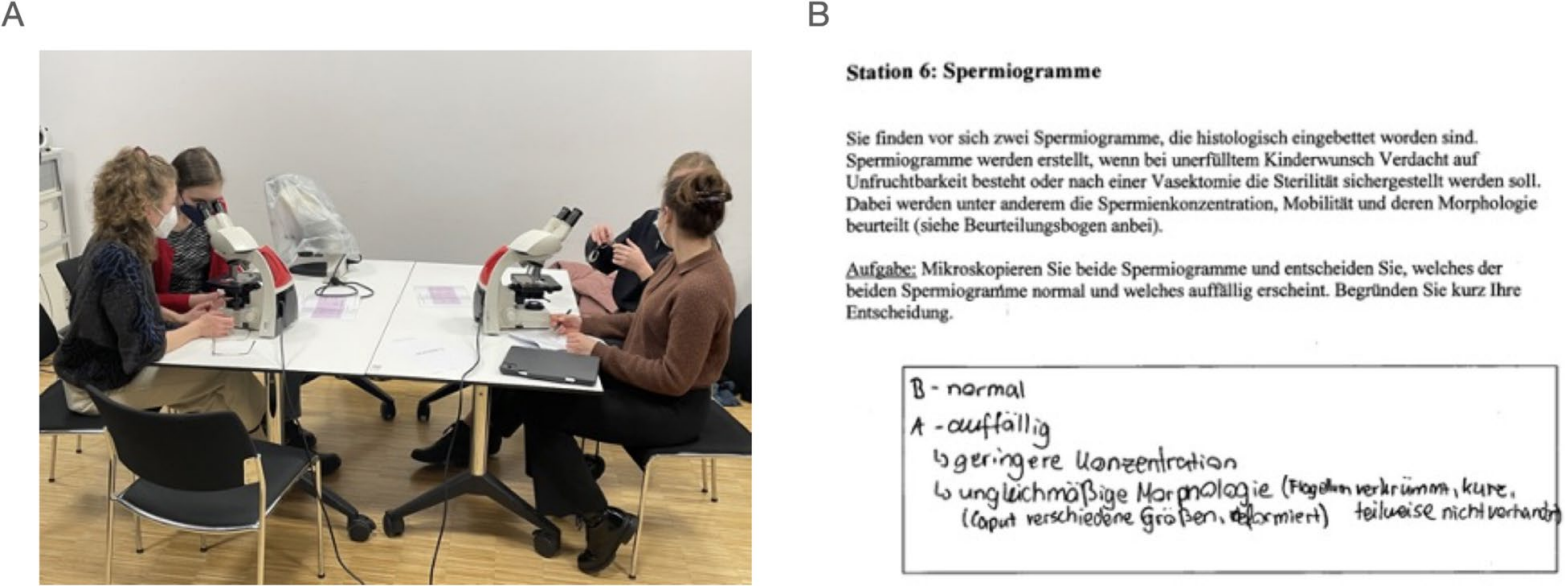
Example of the “Interprofessional Learning Station” six. **A** The goal was to differentiate between healthy and unhealthy sperms based on various criteria (i.e., concentration, morphology, etc.) within a certain time limit. Medical and midwifery students both had to assess the specimen under the microscope in pairs, exchange information, and document their findings on the worksheet. **B** The interprofessional learning objective was to conduct a joint documentation as part of an interprofessional collaboration, while the anatomical learning objective was to differentiate and evaluate healthy and unhealthy sperms

The different interprofessional training stations required students to exchange and discuss skills and knowledge across various areas of anatomy, patient care and medical fundamentals (Fig. 2). Students from both disciplines actively built new knowledge to complete tasks. Learning did not occur through the institute lecturers’ teaching, as their role was to merely oversee and moderate the learning stations. Due to differing curricula, either the midwifery or the medical students’ knowledge levels could determine success in specific tasks. Their clinical and academic responsibilities could be demonstrated to the other profession without any bias. Both parties were able to score equally within the tandem, fostering equitable learning. For instance, midwifery students had limited exposure to dissection rooms, while medical students regularly dissected cadavers. This experience helped medical students alleviate the midwives’ apprehension regarding body donations and instill the necessary respectful ethical behavior.

In the dissection room, ethical work with body donors in interprofessional teams was a primary focus. For instance, station nine concentrated on fetal relics in a cadaver, such as the ligamentum teres hepatis and ductus arteriosus. Students acquired knowledge in an authentic learning environment, termed “situated learning”. This approach enabled students to visualize structures and engage in discussions within their interprofessional tandems. Station seven challenged participants to memorize and identify muscles and structures on 3D pelvic floor models, emphasizing joint documentation as part of interprofessional collaboration. Station ten tasked students with drawing and labeling the anatomical differences between female and male pelvic floors, employing the “learning with drawing” strategy. Furthermore, the students were challenged to create a document that encouraged discussion and agreement within interprofessional tandems. Station eleven tested embryological knowledge by having students identify structures on embryonic models of the 7th, 15th and 25th development days. Station twelve required tandems to name sex differences in male and female pelvic construction using male and female skeletons. Both, station eleven and twelve, employed “situated learning”, prompting students to exchange their individual skills and knowledge to reach a common conclusion.

Theoretical learning stations compelled participants to collaborate in solving tasks through knowledge exchange, discussion and evaluation. For example, in station one, students applied female reproductive system anatomy to address an ectopic pregnancy case. Station six involved comparing and evaluating healthy and unhealthy sperms, also utilizing “situated learning”. Station two fostered mutual appreciation for each other’s expertise by requiring tandems to create a fictional conversation to accompany and educate a woman after childbirth.

### Study participants

The participating students were recruited through regular courses in the medical and midwifery science degree programs. The idea of the project was very well received. According to the students’ feedback, only scheduling difficulties prevented some students from participating.

### Measurements

#### Interprofessional socialization and valuing scale (ISVS)

The Interprofessional Socialization and Valuing Scale (ISVS) was developed by King et al. (2010) [18] to evaluate the beliefs, behaviors, and attitudes that underlie interprofessional socialization and collaborative practice in health care settings. The scale is closely related to the extended professional identity scale of Reinders et al. (2020) [19]. The ISVS is a 24-item self-report measure with a 7-point scale used to assess the extent of the shift towards collaborative care in health care settings [20]. A sample item was: “I have a better appreciation for using a common language across the health professionals in a team”. Three subscales can be identified: The ability to work with others, as well as value and comfort of working with others. The reliability of the scale was deemed adequate (Cronbach’s *α* = 0.67).

#### Maclead clark professional identity scale (MCPIS)

In order to control for socially desirable response behavior (i.e., the tendency of the students to adjust their answers to the perceived expectations of the study), we used a second scale, which measured a closely related but nevertheless different construct. For this purpose, we used the MCPIS, which we assumed to be a subordinate dimension of interprofessional identity [19]. The MCPIS is a validated instrument developed for the measurement of the professional identity of health and social care students from different professions. This scale was first developed by Adams and colleagues (2006) [1] for the measurement of PI in first-year students before the beginning of their training. We used the professional identity subscale of the questionnaire, which consists of 9 items measured on a five-point Likert scale. A sample item was: “I feel like I am a member of this profession.” The reliability of the scale was deemed adequate (Cronbach’s *α* = 0.73).

#### Qualitative essays and content analysis

For methodological triangulation purposes and to avoid a common-method bias, we adapted the professional identity formation essay by Kalet et al. (2017) [21]. The 9 free-text items addressed mainly *mono*professional identity, e.g. with the sense of belonging to one’s own professional group, however, we specifically screened for answers related to *inter*professional identity, for example with regard to the perception of cooperation within the training. A sample item was: “What does being a member of the medical profession mean to you? How did you come to this understanding?”. The analysis was conducted using qualitative content analysis according to Mayring (2014) [21], and encompassed the following steps: developing a codebook, categorizing data, coding, ensuring reliability among raters via intraclass correlation coefficient (ICC) calculations, creating supracategories and quantifying qualitative data for comprehensive analysis.

For the evaluation of the free-text answers and to analyze the given content systematically, we operated with a category system, a codebook, that attempted to bundle the answers of the study participants into common categories. The utilization of the codebook allowed a more refined, focused and efficient analysis of the raw data in subsequent reads. After many iterations of the preliminary codebook, the final version was agreed upon by all four members of the research team. The iterative process of refining the codebook and the consensus among research team members ensured the validity of the categories used. In a similar inductive way, we assigned supracategories.

Subsequently, the same members independently read all the given answers again to assign the individual responses of the study participants to the defined categories. To evaluate the consistency among raters, we computed the Intraclass Correlation Coefficient (ICC) employing a two-way random effects model. The ICC for individual measures stood at 0.86, while the ICC for average measures reached 0.95, signifying good and excellent reliability, respectively (Koo and BPS). Finally, the frequencies of the responses received in each case were compared, and a relative frequency was calculated. This was done in order to be able to compare frequency distributions independently of the sample size. In this way, the qualitative answers could be quantified to enable a much more comprehensive analysis of a problem as well as to get a more comprehensive understanding.

#### Follow-up

A follow-up was conducted 12 weeks after the interprofessional training. The “long-term” effects of the training on IPI were evaluated using the same scales, i.e., ISVS and MCPIS, as well as intergroup contact-related items. We asked if they would participate in interprofessional trainings again and in what kind of situations they refer back to their training experience in their daily lives (“preparations for anatomy tests, internship, awareness of interprofessional communication when team working in the hospital”). In addition, we created an item ranging from 1 to 10 (1 = *no contact*, 10 = *daily contact*) to assess the frequency of contact among students from different professions. Additionally, participants were asked to provide examples of places and platforms where they interact with each other to validate their responses.

### Analysis

Statistical analyses were conducted using the software SPSS v. 29.0.0.0 (IBM SPSS Statistics). As the MCPIS and ISVS scales were administered to participants before and after the intervention, the quantitative analysis pipeline for the provided results involved conducting an ANOVA with dependent samples, where time was considered a within-subject, and intervention (i.e., the training) a between-subject factor, to compare pre- and post-test scores on the scales. All statistics were performed under a significance value of alpha = 0.05, and the results are reported with a two-sided *P*-value. The statistical testing results were specified by an effect size with η^2^ = 0.01 considered a small effect, η^2^ = 0.06 a moderate effect, and η^2^ = 0.14 a strong effect. For the comparison of frequency distributions, regardless of the sample size from both professions, the relative frequency for each profession was calculated (Table 1). This process of converting the qualitative answers into quantitative values aimed to provide a more objective evaluation and allowed a systematic and objective analysis of the collected qualitative data.

**Table 1:** Results of the qualitative content analysis prior to the interprofessional training to explore the professional identities of medical and midwifery students

Code	Main themes	Examples	Relative frequency (%)	
			Midwifery students	Medical students
**Professional understanding**				
A1	Taking responsibility	responsibility for family and friends in the private sphere/patients in the hospital and doctor’s practice.	2.27	4.83
A2	Part of a system	health system; team at the hospital	1.13	1.14
A3	Social function	assistance in everyday life; social commitment	4.22	4.98
A4	Self fulfillment	guarantee of a better quality of life; privilege to know and to exercise	0.81	1.14
**Personal career expectations**				
B1	Professional competence	Expertise	5.19	5.26
B2	Personal competence	autonomy; resilience; blunting	2.43	2.27
B3	Social competence	interprofessional communication, empathy	4.54	5.26
B4	Motivational attitudes	diligence, perseverance, joy	3.56	1.42
**External career expectations**				
C1	Professional competence	theoretical and practical	4.54	2.85
C2	Personal competence	resilience; stress resistance; personality building; responsibility	4.70	4.27
C3	Social competence	attentiveness to others; teamwork (interprofessional)	6.48	5.69
C4	Motivational attitudes	discipline; perseverance; willingness to continue education	2.59	2.56
**Potential for conflicts**				
D1	Career	lack of staff, communication with patients and colleagues; disagreements between professional groups/with patients/regarding treatment methods; misunderstanding; shift work; non defining of clear boundaries	7.13	5.41
D2	Private life	work-life balance; family; leisure time/hobbies; separating work and private life; stress management	4.86	6.26
**Unfulfilled personal expectations**				
E1	Personal consequences	self-doubt; lack of motivation; fear of failure; mental health	5.67	4.98
E2	Professional consequences	endangering third parties; unsuitable for the profession; questioning the profession due to bad experiences	4.05	4.27
**Unfulfilled patient expectations**				
F1	Personal consequences	self-doubt; overestimating oneself in the case of error avoidance/accomplishment; loss of trust; damage of reputation	3.08	4.98
F2	Professional consequences	doing harm, when in case of feeling unwell	5.35	2.70
**Unfulfilled societal expectations**				
G1	Personal consequences	doubts about own competence	1.94	4.13
G2	Professional consequences	damage to the image of the profession; loss of confidence; impact on other generations/against academisation of midwifery	4.22	2.42
**Professional role models**				
H1	Professional competence	individually adapted treatment	4.54	4.13
H2	Personal competence	resilience; clear demarcation between private and professional life	2.92	4.55
H3	Social competence	teamwork and communication (interprofessional; with patients)	6.48	6.83
H4	Motivational attitudes	joy; motivation	0.97	0.57
**Professional upbringing**				
I1	Career	study; exchange with fellow students/members of the profession/patients; positive/negative examples; dissection course; internships	5.02	6.54
I2	Private life	society; social organisations	1.30	0.57

## Results

### Participants

In total, 42 students were enrolled in this curriculum: 24 first-semester students of midwifery sciences and 18 s-semester students of medicine were recruited. A total of 15 (63%) out of the 24 eligible midwifery students answered the survey before the training, and 13 (54%) answered the survey immediately after the training. All 18 medical students (100%) answered the survey before the activity and 17 afterward (94%). Seven midwifery students (29%) and eight medical students (44%) participated in a follow-up 12 weeks after the training.

The majority of the participants in both professions identified themselves as female: 93% of the midwifery students identified themselves as female (*n* = 14), 7% as non-binary (*n* = 1). Among the medical students, 89% of the participants were female (*n* = 16), 11.1% male (*n* = 2). The median age of the midwifery students was 20.47 years (*SD* = 2.32, *range =* 18–28 years), the median age of the medical students was 20.50 years (*SD* = 1.25, *range* = 19–24). Both groups did not significantly differ in age, 𝛘^2^(6) = 4.64, *P* = .591. One student from each group of professions already had a completed professional education.

### Exploring interprofessional identity prior to interprofessional training

Free-text responses before training were analyzed qualitatively to explore the (inter)professional identities of midwifery and medical students. Social competence, i.e., interprofessional teamwork and collaboration, emerged prominently as a career expectation in both personal (4.54% vs. 5.26%) and external career contexts (6.48% vs. 5.69%). Participants similarly placed great value on social competence when considering their professional role models, as evident in category H3 (6.48% vs. 6.83%). Interestingly, the lack of social competences, particularly working within interprofessional teams, were identified as potential sources of conflict for participants (7.13% vs. 5.41%). These findings point to the need for interprofessional training programs that specifically address the development of social competence and teamwork skills.

### Short-term effects of an ultra-brief training on interprofessional socialization, valuing and professional identity

**Fig. 3:**
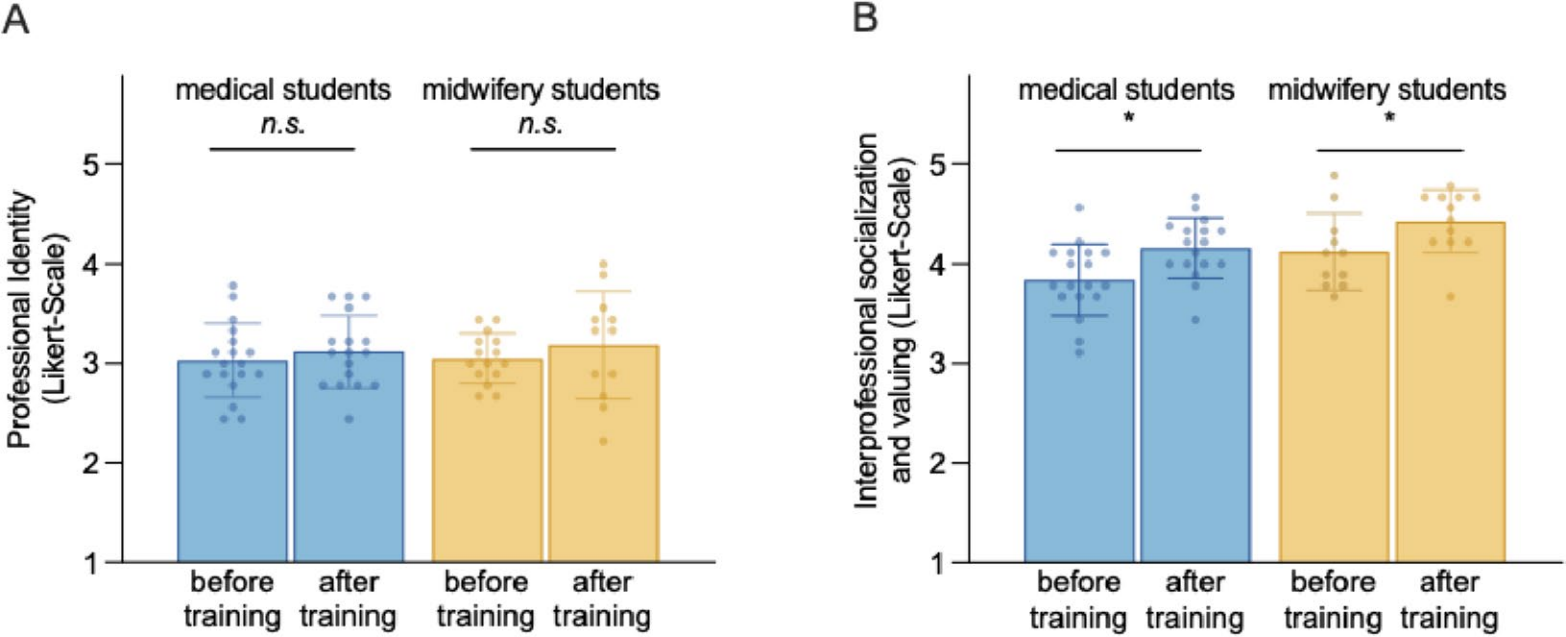
Short-term effects of an ultra-brief (4-hour) interprofessional anatomy training on professional identity formation (MCPIS) (**A**) and interprofessional valuing (ISVS) of medical and midwifery students (**B**). * = *P* < .01

Before training, the ISVS score was lower for medical students (3.87 ± 0.33) than for midwifery students (4.10 ± 0.32). This number increased to 4.13 ± 0.3 resp. 4.43 ± 0.33 after the training (Fig. 3b). The ISVS showed a significant improvement with a strong effect size for both medical and midwifery students, *mean* difference = 0.303, [95% CI: 0.120, 0.486], *P* < .001, η² = 0.171. However, it was noticeable that midwifery students had slightly higher values for the MCPIS compared to medical students, 4.20 resp. 4.31 vs. 3.86 and 3.88, yet the results of the ANOVA showed no pre-post differences in MCPIS scores before and after the training, *mean* difference = 0.033, [95% CI: -0.236, 0.249], *P* = .789 (Fig. 3a). To conclude, the ultra-brief anatomy training fostered interprofessional socialization and valued both midwifery and medical students without affecting their uniprofessional identities.

### Themes identified in free-text responses after training

Table 2 presents the analysis of free-text responses provided by participants after completing the interprofessional training program. These responses were coded into different themes, and the relative frequency of each theme was calculated, with a distinction made between midwifery and medical students’ perspectives. The analysis of free-text responses revealed a generally positive outlook on the interprofessional training, emphasizing the value of professional collaboration, personal growth, improved social relationships, and a more positive perspective on each other’s professions among participants from both midwifery and medical backgrounds. The professional and social dimensions of collaboration were frequently mentioned categories, and participants from both groups valued the harmonious and helpful interactions during the training. Notably, these responses revealed distinct differences between midwifery and medical students in their reflections on the training program. For example, while both groups recognized the social dimension of the training, a higher percentage of medical students (13.81%) expressed increased appreciation and respect for the other profession compared to midwifery students (5.33%).

**Table 2:** Key themes in free-text responses after ultra-brief interprofessional training: Relative frequencies for midwifery and medical students are shown

Code	Main themes	Examples	Relative frequency (%)	
			Midwifery	Medical
**Experiences during the training**				
J1	Positive	interesting; instructive; supportive	25.18	22.38
J2	Neutral	different approaches	3.82	3.33
J3	Negative		0.00	0.96
**Collaboration during the training**				
K1	Professional	instructive; complementing each other’s knowledge	15.27	12.86
K2	Personal	personal exchange	1.53	0.47
K3	Social	harmonious; at eye level; helpful; prejudiced	17.56	14.28
K4	Motivational	exciting; open-minded; pleasant	13.74	10.96
**Perspective on the other profession**				
L1	Professional	recognition of the need for cooperation/similarities of the study programs; more realistic image of the other profession; confirmation of positive/negative image	15.27	12.38
L2	Personal		0.00	0.96
L3	Social	more appreciation and respect; hopeful picture for cooperation in the future	5.33	13.81
L4	Motivational	desire for further exchanges against prejudices; amazement at the knowledge of the others	2.29	7.61

### Long-term effects on interprofessional socialization and valuing

**Fig. 4:**
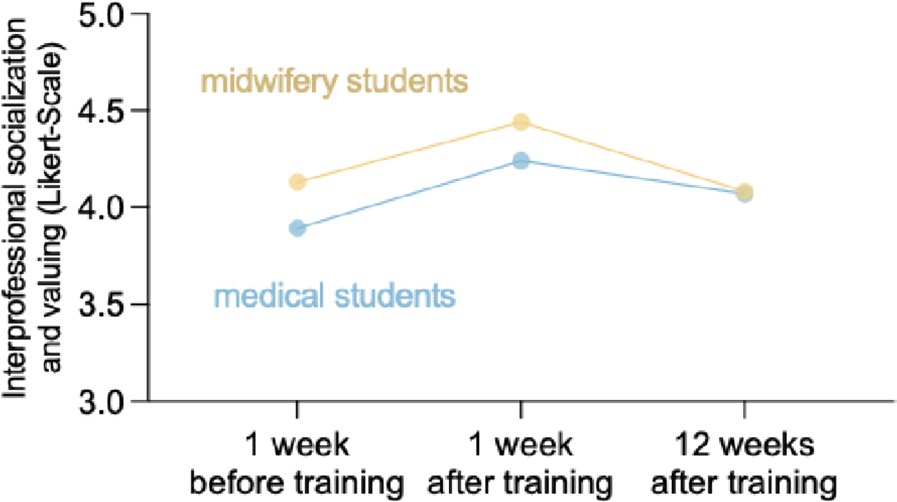
Long-term effects of the ultra-brief interprofessional anatomy training on interprofessional valuing (ISVS) of medical and midwifery students

To assess the stability of the effect observed on interprofessional socialization and valuing, we re-examined a subgroup of students’ 12 weeks after the training (Fig. 4). Similarly, their results indicate an increase in ISVS values from 3.89 (*SD* = 0.17) and 4.13 (*SD* = 0.36) before training to 4.24 (*SD* = 0.28) and 4.44 (*SD* = 0.37) after training. However, 12 weeks after training, we observed a decline of the ISVS back to nearly baseline levels of 4.07 (*SD* = 0.44) and 4.08 (*SD* = 0.42). To conclude, the short-term effects observed after the training were not sustainable after 12 weeks.

The intergroup contact rate for medical students was 2.63 (SD = 1.69), and 3.29 (SD = 2.63) for midwifery students. Common meeting places for both groups included the library (14.29%), university sports facilities (10%), the train station (10%), WhatsApp (14.29%), the cafeteria (42.86%), and university buildings (28.57%).

All participants expressed their willingness to attend the training again, highlighting the enjoyable nature of the experience, the opportunity to gain different perspectives, the ability to learn from one another, the establishment of new connections, preparation for their future careers, and the exchange of ideas. For instance, a medical student noted, “The exchange allowed us to have meaningful discussions and share common interests.” A midwifery student also pointed out that students from both disciplines experienced similar challenges in their education, and this training provided a valuable overview of topics they had already covered in class. This was exemplified by a midwifery student who said, “I realized that both professions encounter comparable difficulties, and we can support each other.”

Medical students expressed their intention to apply the training experience when studying for anatomy exams, during clinical internships, and in their interactions and teamwork within the hospital setting. Likewise, midwifery students found value in recalling their training experience during their daily duties in the hospital, exam preparations, and even in the cafeteria. As one midwifery student put it, “It helped me solidify my knowledge”. In conclusion, despite the quantitative data showing a decrease after 12 weeks, students reported positive outcomes in terms of intergroup interactions and their ability to apply these skills in clinical settings.

## Discussion

Although the importance of interprofessionalism in healthcare professions is recognized, the implementation of appropriate training formats remains a challenge for health profession curricula. The purpose of the current study was to investigate the potential of a theory-derived ultra-brief (i.e., 4 h) interprofessional training to promote key elements of interprofessionalism. The training showed strong effects on both professions in terms of interprofessional socialization and valuing (H1). The follow-up data indicated that these short-term effects did not remain stable over a period of three months (H3), yet positive intergroup contact was still evident (H2). Qualitative data suggested that the participants may have had transformative social experiences during their training, indicating a first step towards establishing an interprofessional identity. We believe that the results of this study provide an important contribution to the current discussion on the implementation of IPE curricula [22]. In the following, we evaluate our curriculum against the backdrop of existing literature, discuss potential strengths and weaknesses, and derive recommendations for future projects.

Bringing together members of different social groups is considered one of the most promising methods for improving relationships between these groups [23]. However, it is important to emphasize that intergroup contact between different groups per se does not guarantee an improvement in relations; it can also reinforce prejudices. The current ultra-brief interprofessional training, despite its brevity, demonstrated positive effects on medical and midwifery students. Importantly, the quantitative and qualitative data indicate that both professions benefited equally from the training format, and there were no indications of asymmetric interactions. Likewise, we observed no negative stress-inducing events associated with the training, which could have resulted in a negative perception of the other profession [1]. 

Of particular note is the strong effect size of the intervention on interprofessional socialization and valuing, with η² = 0.171 (Fig. 3b). We attribute this effect particularly to incorporating several socio-constructivist learning theories into the curriculum design. For example, we placed considerable emphasis on the necessity of prior knowledge from both professions to successfully address the tasks. This *positive interdependence*, widely acknowledged in the literature as a critical foundation for successful collaboration [16], led to the inclusion of both tandem partners. In this way, the participants encountered each other on the same level, getting to know the respective team members on a personal level. Working collaboratively in the team allowed them to mutually support one another in different tasks while learning about the boundaries of their own profession. Concurrently, the competitive setting among the interprofessional tandems resulted in a shift in the identification subject from one’s own profession (“we as the medical students”) towards an interprofessional team (“we as an interprofessional tandem”) [24]. Together, we suggest that forthcoming curricula leverage the theoretical insights as a foundation when developing new educational formats.

Overall, we see early contact between professions as an excellent opportunity to break down barriers early on and establish a mutual understanding of roles. Following Khalili et al.‘s three-stage model [13], this early exchange could be an effective means of overcoming barriers and practicing interprofessional collaboration, ultimately leading to the formation of a dual identity. The early implementation of such training formats allows for the strengthening of identity-forming facets in a protected environment and a gradual approach to a new identity. This prevents the development of dysfunctional role perceptions and stereotypical notions about other professions in uniprofessional silos which constantly reinforce themselves when unsupervised and can lead to conflicts in future professional environments [22]. We also argue that it is more difficult and time-consuming to change an established dysfunctional identity later on than to support interprofessional socialization early on. However, the follow-up results warn against understanding such trainings as singular events, as the effects on interprofessional socialization seem to diminish over time. Therefore, we recommend anchoring such training formats in longitudinal curricula.

The current training was performed with medical and midwifery students, however, embedding such training formats in the core anatomy curriculum [25], in our experience, can be flexibly expanded to other health professions. Since most health professions begin their first semesters with anatomy lessons, both anatomical and interprofessional learning objectives can be cleverly combined. This not only avoids cutting valuable time from the anatomical core curriculum – a subject that has been heavily affected by curricular time reductions in recent years [26] – but also presents an excellent opportunity to allocate additional curricular time for interprofessional training formats. Additionally, we have found that the subject of Anatomy lends itself well to create various collaborative learning formats [27]. This includes, for example, working with body donors, which requires interaction between professions considering ethical principles, a core element of IPE [28]. Similarly, the vertical integration of clinical-obstetric content into anatomy is feasible and has allowed us to construct authentic cases that interprofessional tandems must tackle together. In our curriculum, this included clinical-anatomical cases on ectopic pregnancies or the examination of spermiograms using real microscopes (see Table 3). For future sessions, we plan to set up ultrasound devices and assign specific examination tasks to interprofessional tandems [29]. We believe that such authentic exercises will lower the threshold for future interprofessional collaboration, as the high authenticity of the stations results in socio-constructivist and situated learning [30, 31]. Situated learning theory emphasizes authentic learning environments as particularly useful to promote effective collaboration and (inter)professional identity formation (“feeling like an interprofessional team solving a real task”) [2, 3]. To further support students’ interprofessional identity, we plan to recruit the current study participants as interprofessional peer-teachers for future versions of this curriculum, who may serve as positive role models for interprofessional collaboration and communication.

**Table 3:** Socioconstructivist interprofessional learning stations in anatomy for first-year medical and midwifery students, 4-hour block training in interprofessional tandems

Station number	Station name	Anatomical learning objectives	Interprofessional learning objectives	Didactic strategy	Materials used	Points achieved, mean (SD)
**Theoretical stations – Multimedia room (12 min. per station)**						
1	Ectopic pregnancy	Apply the anatomy of the female reproduction to solve an authentic case of an ectopic pregnancy	Use interprofessional communication to solve an authentic problem	Case-based learning	Pen-and-pencil	6.12 (1.73)
2	Postpartum	Formulate a fictional conversation to accompany and educate a woman after childbirth	Value the midwives’ area of responsibility and the complexity of their profession	Simulation	Pen-and-pencil	5.53 (1.50)
3	Hormone memory	Examine endocrine hormones and allocate them to their functions	Distribute tasks self-directed in an interprofessional team	Gamification	Memory cards	5.18 (2.04)
4	Sectio cesarea	Analyze the anatomical structures in a sectio cesarea	Communicate adequately as part of an interprofessional team	Case-based learning	Tablets with movie clips	4.18 (1.47)
5	Emergency room	Apply anatomical knowledge to simulate clinical reasoning in the emergency room	Value the medical students’ responsibility and the complexity of their profession	Simulation	Laptops with an ER simulation software	1.06 (0.90)
6	Spermiogram	Differentiate and evaluate healthy and unhealthy sperms	Conduct a joint documentation as part of an interprofessional collaboration	Situated learning	Microscopes and sperm specimen	3.35 (2.06)
**Practical stations – Dissection hall (12 min. per station)**						
7	Pelvic floor	Memorize and repeat the muscles and structures of the pelvis and locate them on 3D pelvic floor models	Conduct a documentation as part of an interprofessional collaboration	3D models	Male and female pelvis models	6.88 (2.74)
8	Lower abdomen	Identify and compare the anatomy of the lower abdomen in a female and male cadaver	Work ethically with body donors in interprofessional teams	Situated learning	Cadaver and flags to mark the structures to be designated	5.03 (0.98)
9	Fetal relics	Name and find fetal relics in cadavers	Work ethically with body donors in interprofessional teams	Situated learning	Cadaver and flags to mark the structures to be designated	4.58 (2.18)
10	Pelvic floor	Draw, label and name the differences of the female and male pelvic floor	Conduct a documentation as part of an interprofessional collaboration	Learning with drawing	Median sagittal section of cadavers	7.76 (5.13)
11	Embryonic period	Identify structures on embryonic models of the 7th, 15th and 25th developmental day	Distribute tasks self-directed in an interprofessional team	Situated learning	Three numbered models	4.76 (2.80)
12	Sexual dimorphism of the bony pelvis	Name all the sex differences in the construction of the pelvis in male and female skeletons	Distribute tasks self-directed in an interprofessional team	Situated learning	Male and female skeletons	4.62 (1.87)

### Limitations

Several limitations must be acknowledged. First, all the instruments that were used in this study relied on the self-reported data of the students, which may have inflated correlations due to shared method variance, such as tendencies toward socially desirable responses or further responding tendencies. To estimate the extent of socially desirable responses, we used a closely related uniprofessional identity scale as a control construct. Still, further research may advance the implementation of behavior-oriented measures of interprofessional socialization, or IPI, even though there are only a few studies that consider the objective assessment of such measures. Second, study participation was voluntary, so participating students may not be representative of the whole cohort. Finally, we lost some participants in the follow-up, so the follow-up data must be interpreted cautiously as they represent only a subset of the study population.

## Conclusion

“Working together has given me hope, that as the next generation of doctors and midwives, we can break the old hierarchy and work together as a functioning team.” In essence, this participant’s perspective not only acknowledges the transformative potential of an ultra-brief interprofessional training in anatomy but also advocates for a paradigm shift in educational approaches. By emphasizing the importance of teamwork and breaking down hierarchical structures early in the educational journey, we may cultivate a generation of healthcare professionals who are not only proficient in their respective fields, but also inherently collaborative and well-prepared to meet the complex challenges of modern healthcare.

## Data Availability

The datasets used and/or analyzed during the current study available from the corresponding author on reasonable request.
